# Maternal-zygotic knockout reveals a critical role of Cdx2 in the morula to blastocyst transition

**DOI:** 10.1016/j.ydbio.2014.12.004

**Published:** 2015-02-15

**Authors:** Agnieszka Jedrusik, Andy Cox, Krzysztof Wicher, David M. Glover, Magdalena Zernicka-Goetz

**Affiliations:** a Department of Physiology, Development and Neuroscience, University of Cambridge, Downing Site, Cambridge CB2 3DY, UK; b Department of Genetics, University of Cambridge, Downing Site, Cambridge CB2 3EH, UK

**Keywords:** Mouse embryo, Cdx2, Maternal-zygotic knockout, Trophectoderm

## Abstract

The first lineage segregation in the mouse embryo generates the inner cell mass (ICM), which gives rise to the pluripotent epiblast and therefore the future embryo, and the trophectoderm (TE), which will build the placenta. The TE lineage depends on the transcription factor Cdx2. However, when Cdx2 first starts to act remains unclear. Embryos with zygotic deletion of *Cdx2* develop normally until the late blastocyst stage leading to the conclusion that *Cdx2* is important for the maintenance but not specification of the TE. In contrast, down-regulation of *Cdx2* transcripts from the early embryo stage results in defects in TE specification before the blastocyst stage. Here, to unambiguously address at which developmental stage Cdx2 becomes first required, we genetically deleted *Cdx2* from the oocyte stage using a Zp3-Cre/loxP strategy. Careful assessment of a large cohort of *Cdx2* maternal-zygotic null embryos, all individually filmed, examined and genotyped, reveals an earlier lethal phenotype than observed in *Cdx2* zygotic null embryos that develop until the late blastocyst stage. The developmental failure of *Cdx2* maternal-zygotic null embryos is associated with cell death and failure of TE specification, starting at the morula stage. These results indicate that Cdx2 is important for the correct specification of TE from the morula stage onwards and that both maternal and zygotic pools of Cdx2 are required for correct pre-implantation embryogenesis.

## Introduction

Prior to zygotic genome activation (ZGA), embryonic development is dependent on maternally inherited RNAs and proteins (Bachvarova and De Leon, 1980; Braude et al., 1979; Cascio and Wassarman, 1982; Van Blerkom, 1981). In the mouse, there are two waves of ZGA: a minor one at the late zygote stage and a major one at the 2-cell stage (Flach et al., 1982; Latham and Schultz, 2001). Despite this early ZGA, maternal factors are recognised as essential for embryo viability and also lineage segregation (Keramari et al., 2010; Zuccotti et al., 2011). Maternal factors can either rescue or significantly delay development of the mutant phenotype in zygotic knockout embryos (Avilion et al., 2003; Larue et al., 1994; Reithmacher et al., 1995). It is therefore important to consider the contribution of both maternal and zygotic gene expression to embryo development, particularly at the pre-implantation stages.

The first lineage segregation in the mouse embryo leads to separation of inside and outside cells that occurs in two cell division waves: at the 8–16 cell and 16–32 cell transition (Bischoff et al., 2008; Graham and Deussen, 1978; Johnson and Ziomek, 1982; Pedersen et al., 1986). Inside and outside cells will develop their unique identity due to their differential position and due to asymmetric partitioning of cell polarity proteins, such as Par1, Par3, aPKC, Jam1, Ezrin and transcripts, such as *Cdx2* (Louvet et al., 1996; Nishioka et al., 2009; Skamagki et al., 2013; Tarkowski and Wroblewska, 1967; Thomas et al., 2004; Vinot et al., 2005). Together differential partitioning of key cellular components and differential cell positioning establish the inside–outside asymmetry within the embryo that leads to development of the ICM and TE lineages.

Cdx2 is an essential transcription factor for the development of the mouse embryo at many developmental stages (Gao et al., 2009; Grainger et al., 2010; Morris et al., 2014; Stringer et al., 2012; van Rooijen et al., 2012; Zhao et al., 2014). During pre-implantation development, Cdx2 is essential for the TE lineage, but the stage of development at which Cdx2 plays a role and the processes it controls both remain unclear. Embryos with zygotic deletion of *Cdx2* develop normally until the late blastocyst stage leading to the suggestion that Cdx2 is involved only in maintenance of the TE lineage (Ralston and Rossant, 2008; Strumpf et al., 2005). However, down-regulation of both maternal and zygotic *Cdx2* expression by RNAi or morpholino treatments results in a much earlier phenotype that includes defects in cell polarisation, developmental arrest (Jedrusik et al., 2010) and the abnormal activity of mitochondria (Wu et al., 2010). These studies led to the suggestion that Cdx2 might have two roles in pre-implantation development: first, to ensure appropriate cell polarisation that is critical for TE formation and second, the subsequent maintenance of the TE lineage.

Functionality of maternal Cdx2 was recently questioned as embryos in which maternal *Cdx2* expression was genetically eliminated developed normally (Blij et al., 2012). Here, to address this discrepancy we have genetically ablated both maternal and zygotic *Cdx2* and filmed development of *Cdx2* maternal-zygotic and *Cdx2* maternal and *Cdx2* zygotic knockout embryos side-by-side to compare development to the blastocyst stage. This revealed that embryos deficient for both maternal and zygotic *Cdx2* show significantly reduced developmental potential and increased cell death from the morula stage onwards. The developmental lethality is significantly stronger following depletion of both maternal and zygotic pools of Cdx2, rather than when only maternal or only zygotic Cdx2 are eliminated. Together, these results lead us to conclude that both maternal and zygotic Cdx2 are important for the development of the mouse embryo and that the first stage of development at which Cdx2 plays a role is at the morula stage when specification of the TE first starts.

## Materials and methods

### Mouse strains

**Fig. 1:**
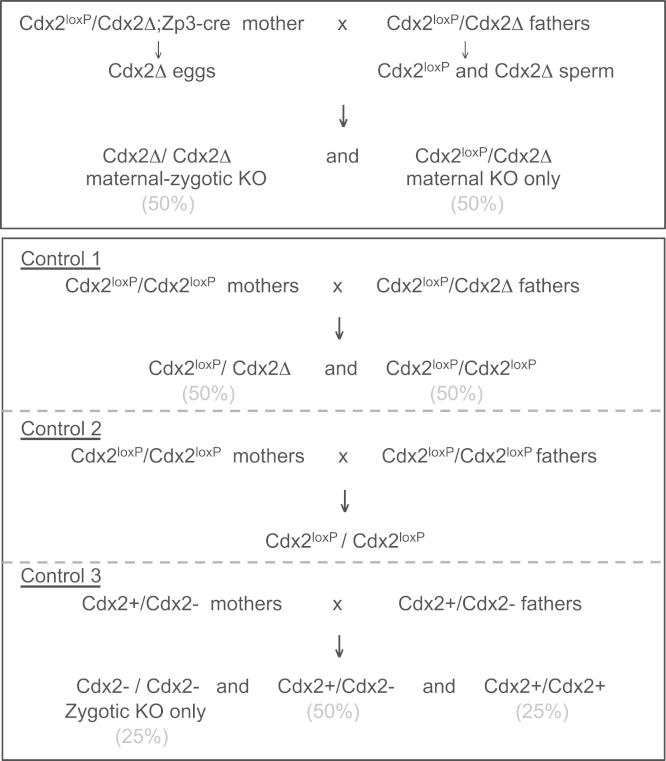
Breeding schemes used to generate *Cdx2* maternal-zygotic knockout and maternal knockout embryos.

To obtain oocytes depleted of maternal *Cdx2*, we used females heterozygous for a *floxed Cdx2* gene (Gao et al., 2009) and a *Cdx2* deletion (Cdx2Δ), and carrying a *Zp3-Cre* transgene (de Vries et al., 2000) (*Cdx2*^*loxP*^/*Cdx2Δ*; *Zp3-Cre* females). These females were mated with *Cdx2*^*loxP*^/*Cdx2Δ* males (Fig. 1). The *Cdx2*^*loxP*^ line (Gao et al., 2009) was a kind gift from Klaus H Kaestner. Complete cleavage of the Cdx2^loxP^ allele in the female germline by Cre recombinase results in 100% of mature oocytes carrying the Cdx2Δ. 50% of the resulting embryos that are *Cdx2Δ*/*Cdx2Δ* are maternal-zygotic knockouts (MZ-KO) and the 50% *Cdx2Δ*/*Cdx2*^*loxP*^ embryos that have a wild-type (but floxed) paternal *Cdx2* allele are heterozygous maternal knockouts (M-KO). Control females were either *Cdx2*^*loxP*^ homozygotes or *Cdx2*^*loxP*^/*Cdx2Δ* heterozygotes and did not carry the *Zp3-Cre* transgene. To obtain embryos depleted of zygotic Cdx2 (Z-KO), mice heterozygous for the *Cdx2* targeted mutation *Cdx2*^*tm1Fbe*^ that we refer to as *Cdx2-* were intercrossed (MGI:1857928) (Chawengsaksophak et al., 1997).

### Embryo recovery, culture and time-lapse microscopy

Mouse embryos were recovered from oviducts of superovulated females (10 IU PMSG, 10 IU hCG; Intervet), collected into M2 medium with 4 mg/ml BSA, and cultured in KSOM supplemented with 4 mg/ml BSA as described before (Jedrusik et al., 2008). To record the development of each individual embryo, embryos were cultured on gridded dishes and filmed by time-lapse microscopy. Imaging was non-invasive and carried out using a wide-field Zeiss microscope with a Hamamatsu Orca ER digital camera and DIC *Z*-stacks were collected at 15 min intervals for 58 h.

To analyse any occurrence of cell death during embryo development, SYTOX fluorescent green nucleic acid stain (Life technologies) was added to the culture medium as previously described (Bedzhov and Zernicka-Goetz, 2014).

### Individual embryo immunofluorescence and genotyping

Individual embryos were removed from the gridded dish upon completion of time-lapse imaging, fixed individually in 4% PFA (4 °C, 12 h) and processed for immunofluorescence as previously described (Plusa et al., 2005). Primary antibodies: mouse anti-Cdx2 (BioGenex; 1:200), rat TROMA-1 anti-cytokeratin-8 (DSHB, Iowa; 1:100); and rabbit anti-Nanog (R&D Systems; 1:200). DNA was stained with Hoechst (Sigma). Images were recorded using a Leica SP5 confocal microscope with a 40× oil objective.

Following immunofluorescence, the genotype of each embryo was determined by PCR. To this end, genomic DNA was extracted from individual embryos in 10 µl extraction/neutralization buffers (Truett et al., 2000). 4.5–5 µl of the lysate was used in PCR reactions using the Fast Cycling PCR Kit (Qiagen) and primers: 5′-GACCGAAGTCTGCAGAACCT and 5′-GGCTCAGGACTTGCTCCTTCA to detect Cdx2 WT and Cdx2 KO alleles; and 5′-AGCCCATTGCTGGACGGAGG and 5′-CCGCTTACCTTGACGCCACA to detect cleaved KO (null) allele.

## Results

### Cdx2 is required for correct development to the blastocyst stage *in vivo*

**Fig. 2:**
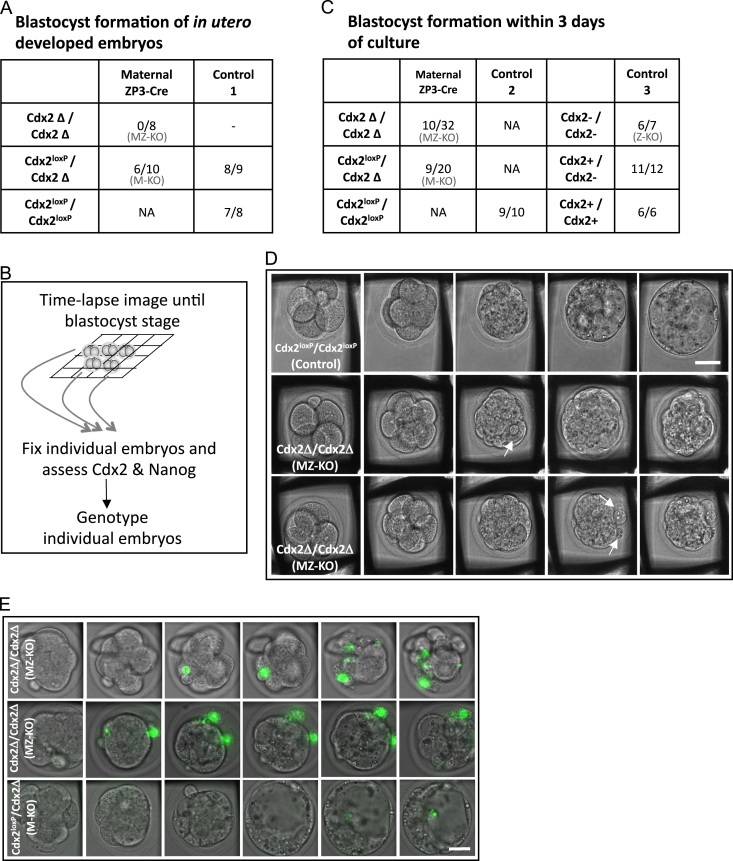
(A) Development of Cdx2 maternal-zygotic null embryos in vivo and in vitro. Summary of data on the frequency of blastocyst formation in embryos developing *in utero.* Experimental embryos are progeny of *Cdx2*^loxP^/*Cdx2*∆; *Zp3-cre* mothers, and control group 1 *Cdx2*^loxP^/ *Cdx2*^loxP^ mothers, in both cases crossed to *Cdx2*^loxP^/*Cdx2*∆ fathers. The embryos were recovered at E3.75. The average number of cells were as follows: control embryos (Cdx2^loxP^/Cdx2^loxP^), 60.5; control embryos (Cdx2^loxP^/Cdx2∆), 63.4; maternal knockout embryos (Cdx2^loxP^/Cdx2∆), 43.4; maternal-zygotic knockout embryos (Cdx2∆/Cdx2∆ ), 29.0. Embryo morphology was assessed immediately after recovery after which embryos were fixed, stained and genotyped. (B) Schematic representation of experimental design for culturing, time-lapse imaging and genotyping individual embryos. (C) Summary of data on the frequency of blastocyst formation in embryos developing *in vitro*. Control group 2 is progeny of *Cdx2*^loxP^ parents; control group 3, are embryos from *Cdx2*+/*−* intercrosses. (D) Examples of embryo development from 4-cell to the blastocyst stage in control (upper panel) and maternal-zygotic knockout (MZ-KO) embryos (lower panels). Dying cells indicated with white arrows. Bar=25 μm. (E) Still images of time-lapse recording of pre-implantation development of *Cdx2* MZ-KO (top two panels) and control (bottom panel) embryos. Dying cells are marked by SYTOX, a fluorescent green cell death reporter. Cell death in MZ-KO embryos is initiated at earlier developmental stages than in control embryos and with significantly higher frequency. Bar=25 μm.

The stage at which Cdx2 is first required for development of the mouse embryo remains unclear because Cdx2 is expressed both maternally and zygotically (Jedrusik et al., 2010; Wu et al., 2010). To address this, we used a genetic approach to deplete maternal and zygotic pools of Cdx2 from the oocyte stage onwards by crossing females heterozygous for a *floxed Cdx2* gene and a *Cdx2* deletion and carrying the *Cre*-*recombinase* gene under control of the *ZP3 promotor* (*Cdx2*^*loxP*^/*Cdx2Δ*; *Zp3-Cre*) with *Cdx2*^*loxP*^/*Cdx2Δ* males (Fig. 1). This cross results in equal proportions of homozygotes for the *Cdx2* deletion and heterozygotes with one deleted copy and one *floxed* copy. We first allowed the embryos to develop *in utero* and recovered them 3.75 days after fertilisation when they should have reached the mature blastocyst stage. To ensure that the embryos were of the highest possible quality, embryos were recovered from naturally mated rather than super-ovulated females. We found that none of the *Cdx2* maternal-zygotic knockout embryos (0 out of 8, 0%) developed to the blastocyst stage (Fig. 2A). This was in contrast to their heterozygous siblings, *Cdx2* maternal-knockout embryos, where 6 out of 10 (60%) embryos developed to the blastocyst stage. In a control group of embryos from *Cdx2*^*loxP*^/*Cdx2*^*loxP*^ mothers lacking the ZP3-Cre transgene, 15 out of 17 (88%) progressed to the blastocyst stage. Thus, deficiency of maternal and zygotic *Cdx2* expression leads to an earlier lethal phenotype than observed for the *Cdx2* zygotic knockout alone when embryos develop normally until the late blastocyst stage (Ralston and Rossant, 2008; Strumpf et al., 2005). These results indicate a role for maternally provided *Cdx2* in development before the blastocyst stage.

### Cdx2 is required for the morula to blastocyst stage transition *in vitro*

To determine the exact stage of the developmental arrest of *Cdx2* maternal-zygotic knockout embryos, we next wished to monitor their progression from the 2-cell stage until the blastocyst stage. In order to observe the developing embryos continuously, we used time-lapse microscopy. Individual embryos were allocated to individual interstices of grids in a culture chamber so that the development of each embryo could be tracked before being genotyped. This allowed us to gather as much information as possible about successive stages of pre-implantation development of all individually genotyped embryos (Fig. 2B). We found that from 32 *Cdx2* maternal-zygotic knockout embryos, 69% arrested development at the morula stage (*Cdx2Δ*/*Cdx2Δ* embryos from *Cdx2*^*loxP*^/*Cdx2Δ*; *Zp3-Cre* mothers; Fig. 2C), in agreement with our *in vivo* experiments above. This was in contrast to *wild-type* embryos of which 90% (*N*=10) developed to the blastocyst stage (Control group 2, progeny of *Cdx2*^*loxP*^/*Cdx2*^*loxP*^ parents; Figs. 2C; *p*=0.002, 2-tail Fisher exact test). We found that 55% (*N*=20) of *Cdx2* maternal only knockout embryos (*Cdx2*^*loxP*^/*Cdx2Δ*) arrested at the morula stage, in contrast to only 14% of *Cdx2* zygotic only knockout embryos obtained by *Cdx2*+/− intercrosses (*Cdx2−*/*−*, Control group 3; *N*=7). Together these results suggest that maternal Cdx2 contributes to normal development from the morula to the blastocyst stage.

### Cell death in maternal and zygotic Cdx2 knockout embryos

In order to understand the developmental failure of *Cdx2* maternal-zygotic knockout embryos, we analysed time-lapse recordings. This revealed that while in *wild-type* embryos cell death does not occur until the late blastocyst stage, in *Cdx2* maternal-zygotic knockout embryos cell death occurs much earlier, at the morula stage (Fig. 2D, arrows). To confirm this observation, we next cultured *Cdx2* maternal and maternal-zygotic knockout embryos in the presence of Sytox, a fluorescent nucleic acid stain which allows detection of cell death *in vivo* by time-lapse microscopy. To ensure that the embryos were of highest possible quality, embryos were recovered from naturally mated rather than super-ovulated *Cdx2*^*loxP*^/*Cdx2Δ*; *Zp3-Cre* females. We analysed development of 25 embryos, from which we were able to genotype 16, from which 10 were *Cdx2* maternal-zygotic knockout embryos and 6 their heterozygous *Cdx2* maternal only knockout siblings (the remaining embryos were lost during procedure or the genotyping result was ambiguous). This revealed that the absence of both maternal and zygotic Cdx2 led to a significantly higher number of dead cells compared to maternal depletion alone (4.1 cells per embryo *versus* 1.7 cells per embryo, respectively; *p*<0.005, 2-tail Fisher exact test). Together these results indicate that total loss of Cdx2 leads to cell death prior to blastocyst formation.

### Cdx2 is required for correct TE specification

**Fig. 3:**
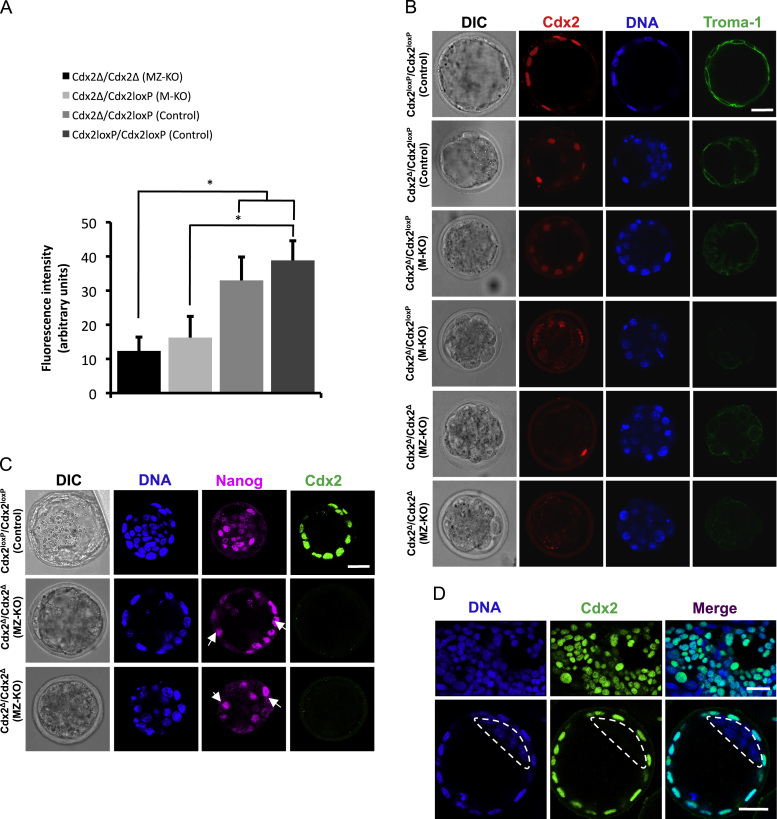
Aberrant trophectoderm specification in embryos lacking maternal and zygotic expression of *Cdx2*. (A) Expression of trophectoderm (TE) specific cytokeratins, recognised by TROMA-1 antibody, is reduced in *Cdx2* MZ-KO (*N*=6) and M-KO (*N*=7) embryos in comparison to both heterozygous (*N*=4) and homozygous (*N*=4) controls allowed to develop for the same time period (4.5 days post-fertilisation). Fluorescent signal intensity was quantified using ImageJ and the pipeline was automated using a macro. Image stacks in the appropriate channels were convolved with a Gaussian blur (*σ*=2) to reduce the effects of detector noise, then thresholded using Li׳s Minimum Cross Entropy thresholding method. This was applied to areas that include all of the labelled membrane while excluding as much background as possible. The mean intensity value of thresholded areas in the stack was calculated. (B) Representative embryos from the quantitative analysis depicted in A. (C) Ectopic expression of Nanog (white arrows) in outside cells of *Cdx2* maternal-zygotic null (*N*=29) but not in control (Cdx2^loxP^) embryos. (D) Positive Cdx2 antibody control showing immunofluorscent detection of Cdx2 in trophoblast stem (TS) cells (top panel), and negative antibody control in E3.5 blastocyst (bottom panel) showing Cdx2 restriction to outer (TE) cells and omission from the ICM (indicated with dashed line).

In order to determine the effect of depletion of both maternal and zygotic *Cdx2* expression on lineage specification, we examined the expression of two lineage markers; the TE marker cytokeratin-8 (recognised by TROMA-1 antibody) and the ICM marker, Nanog. We found that cytokeratin-8 expression was either totally absent or significantly weaker in *Cdx2* maternal-zygotic knockout embryos in comparison to control embryos (Figs. 3A and B). Immunostaining also confirmed the loss of the *Cdx2* TE marker, as expected (Figs. 3A, B, and D). To quantify the decrease in TROMA-1 staining, we have measured the fluorescent signal intensity using ImageJ and an automated macro by which image stacks were convolved with a Gaussian blur to reduce detector noise effects and then thresholded for calculation of the mean intensity. These analyses confirmed a significant decrease in cytokeratin-8 expression in *Cdx2* maternal-zygotic knockout and maternal knockout embryos in comparison to *Cdx2Δ*/*Cdx2*^*loxP*^ and *Cdx2*^*loxP*^/*Cdx2*^*loxP*^ control embryos that developed for the same period of time (*p*<0.05, Student׳s *t*-test). Immunofluorescence for the ICM marker Nanog in *Cdx2* maternal-zygotic knockout embryos revealed its ectopic expression in outside cells (Fig. 3C), in agreement with the role of Cdx2 in down-regulating pluripotency genes. These results indicate that Cdx2 is important for the specification of the TE lineage.

## Discussion

To address when Cdx2 function is required in early mouse development we have eliminated both maternal and zygotic *Cdx2* expression to determine if this would result in an earlier phenotype than previously reported for *Cdx2* zygotic null embryos, which progress normally to the blastocyst stage (Ralston and Rossant, 2008; Strumpf et al., 2005). To this end, we have used a ZP3-cre/loxP strategy that eliminates *Cdx2* from the oocyte and time-lapse imaging to record individual embryos throughout pre-implantation development. This revealed that *Cdx2* maternal-zygotic null embryos show significantly reduced developmental potential to the blastocyst stage, increased cell death and failure of the specification of the TE lineage. This lethal phenotype of *Cdx2* maternal-zygotic null embryos occurred irrespective of whether embryos were allowed to develop *in vivo* or *in vitro*. This leads us to conclude that Cdx2 is important for correct development of the pre-implantation embryo due to a role in the specification of TE lineage from the morula stage.

The findings we report here differ from the conclusion of a study examining the consequences of depleting maternal *Cdx2* (Blij et al., 2012). It is unlikely that this difference reflects the effects of embryo culture conditions because we observe the same developmental lethality whether embryos develop *in vivo* or *in vitro*. It is possible that differences in the methods of analysis contributed to the apparent difference in outcome. In assessing developmental potential of *Cdx2* maternal knockout embryos, Blij et al. (2012) reported no difference in litter size between wild-type females and maternal knockout females mated to wild-type males (7.3±1.5 and 7.0±0.5, respectively). By contrast, we found that 40% (*N*=10) of embryos deficient for only maternal *Cdx2*, and yet having zygotic expression of *Cdx2*, failed to develop to the blastocyst stage, compared with only 12% (*N*=17) of a control group within the same period of time. When the contribution of both maternal and zygotic *Cdx2* were depleted (*N*=8), we found that none of the embryos developing *in vivo* reached the blastocyst stage.

Another possibility is the number of embryos examined. Blij et al. (2012) cultured 11 *Cdx2* maternal-zygotic null and 10 *Cdx2* zygotic null embryos together and reported no differences in their development from the single cell to the blastocyst stage. We made time-lapse recordings of 32 individual *Cdx2* maternal-zygotic and 20 individual maternal null embryos. This revealed that the majority (69%, *N*=32) of the *Cdx2* maternal-zygotic null and 55% (*N*=20) of the maternal null embryos did not reach the blastocyst stage within 3 days of culture starting from the 2-cell stage. In comparison, only 15% of *Cdx2* zygotic null embryos (*N*=7) and 7% of control group embryos (*N*=28) failed to reach the blastocyst stage within the same period of time. These results indicate that Cdx2 is important for efficient development to the blastocyst stage.

Our time-lapse experiments revealed cell death in the *Cdx2* maternal-zygotic null embryos at the morula stage whereas cell death does not occur at this developmental stage in *wild-type* embryos. We also found that the *Cdx2* maternal-zygotic null embryos showed either absent or significantly reduced expression of the classic TE maker cytokeratin-8 together with expression of the pluripotent marker, Nanog, in outside cells, suggesting failure in TE specification, in agreement with earlier Cdx2 down-regulation studies from the 2-cell stage (Jedrusik et al., 2010). Taken together, the findings we report here provide evidence that total depletion of Cdx2 compromises pre-implantation development, both *in vivo* and *in vitro*, and that this is associated with increased cell death and defects in TE specification starting in morula stage embryos. These results lead us to conclude that *Cdx2* is involved in normal embryonic development of the mouse before the blastocyst stage.

## References

[bib1] Avilion A.A., Nicolis S.K., Pevny L.H., Perez L., Vivian N., Lovell-Badge R. (2003). Multipotent cell lineages in early mouse development depend on SOX2 function. Genes Dev..

[bib2] Bachvarova R., De Leon V. (1980). Polyadenylated RNA of mouse ova and loss of maternal RNA in early development. Dev. Biol..

[bib3] Bedzhov I., Zernicka-Goetz M. (2014). Self-organizing properties of mouse pluripotent cells initiate morphogenesis upon implantation. Cell.

[bib4] Bischoff M., Parfitt D.E., Zernicka-Goetz M. (2008). Formation of the embryonic-abembryonic axis of the mouse blastocyst: relationships between orientation of early cleavage divisions and pattern of symmetric/asymmetric divisions. Development.

[bib5] Blij S., Frum T., Akyol A., Fearon E., Ralston A. (2012). Maternal Cdx2 is dispensable for mouse development. Development.

[bib6] Braude P., Pelham H., Flach G., Lobatto R. (1979). Post-transcriptional control in the early mouse embryo. Nature.

[bib7] Cascio S.M., Wassarman P.M. (1982). Program of early development in the mammal: post-transcriptional control of a class of proteins synthesized by mouse oocytes and early embryos. Dev. Biol..

[bib8] Chawengsaksophak K., James R., Hammod V.E., Kontgen F., Beck F. (1997). Homeostasis and intestinal tumors in Cdx2 mutant mice. Nature.

[bib9] de Vries W.N., Binns L.T., Fancher K.S., Dean J., Moore R., Kemler R., Knowles B.B. (2000). Expression of Cre recombinase in mouse oocytes: a means to study maternal effect genes. Genesis.

[bib10] Flach G., Johnson M.H., Braude P.R., Taylor R.A.S., Bolton V.N. (1982). The transition from maternal to embryonic control in the 2-cell mouse embryo. EMBO J..

[bib11] Gao N., White P., Kaestner K.H. (2009). Establishment of intestinal identity and epithelial-mesenchymal signaling by Cdx2. Dev. Cell.

[bib12] Graham C.F., Deussen Z.A. (1978). Features of cell lineage in preimplantation mouse development. J. Embryol. Exp. Morphol..

[bib13] Grainger S., Savory J.G., Lohnes D. (2010). Cdx2 regulates patterning of the intestinal epithelium. Dev. Biol..

[bib14] Jedrusik A., Bruce A.W., Tan M.H., Leong D.E., Skamagki M., Yao M., Zernicka-Goetz M. (2010). Maternally and zygotically provided Cdx2 have novel and critical roles for early development of the mouse embryo. Dev. Biol..

[bib15] Jedrusik A., Parfitt D.E., Guo G., Skamagki M., Grabarek J.B., Johnson M.H., Robson P., Zernicka-Goetz M. (2008). Role of Cdx2 and cell polarity in cell allocation and specification of trophectoderm and inner cell mass in the mouse embryo. Genes Dev..

[bib16] Johnson M.H., Ziomek C.A. (1982). Cell subpopulations in the late morula and early blastocyst of the mouse. Dev. Biol..

[bib17] Keramari M., Razavi J., Ingman K.A., Patsch C., Edenhofer F., Ward C.M., Kimber S.J. (2010). Sox2 is essential for formation of trophectoderm in the preimplantation embryo. PloS One.

[bib18] Larue L., Ohsugi M., Hirchenhain J., Kemler R. (1994). E-cadherin null mutant embryos fail to form a trophectoderm epithelium. Proc. Natl. Acad. Sci. USA.

[bib19] Latham K.E., Schultz R.M. (2001). Embryonic genome activation. Front. Biosci..

[bib20] Louvet S., Aghion J., Santa-Maria A., Mangeat P., Maro B. (1996). Ezrin becomes restricted to outer cells following asymmetrical divisions in the preimplantation mouse embryo. Dev. Biol..

[bib21] Morris S.A., Cahan P., Li H., Zhao A.M., San Roman A.K., Shivdasani R.A., Collins J.J., Daley G.Q. (2014). Dissecting engineered cell types and enhancing cell fate conversion via CellNet. Cell.

[bib22] Nishioka N., Inoue K., Adachi K., Kiyonari H., Ota M., Ralston A., Yabuta N., Hirahara S., Stephenson R.O., Ogonuki N., Makita R., Kurihara H., Morin-Kensicki E.M., Nojima H., Rossant J., Nakao K., Niwa H., Sasaki H. (2009). The Hippo signaling pathway components Lats and Yap pattern Tead4 activity to distinguish mouse trophectoderm from inner cell mass. Dev. Cell.

[bib23] Pedersen R.A., Wu K., Balakier H. (1986). Origin of the inner cell mass in mouse embryos: cell lineage analysis by microinjection. Dev. Biol..

[bib24] Plusa B., Frankenberg S., Chalmers A., Hadjantonakis A.K., Moore C.A., Papalopulu N., Papaioannou V.E., Glover D.M., Zernicka-Goetz M. (2005). Downregulation of Par3 and aPKC function directs cells towards the ICM in the preimplantation mouse embryo. J. Cell Sci..

[bib25] Ralston A., Rossant J. (2008). Cdx2 acts downstream of cell polarisation to cell-autonomously promote trophectoderm fate in the early mouse embryo. Dev. Biol..

[bib26] Reithmacher D., Brinkmann V., Birchmeier C. (1995). A targeted mutation in the mouse E-cadherin gene results in defective preimplantation development. Proc. Natl. Acad. Sci. USA.

[bib27] Skamagki M., Wicher K.B., Jedrusik A., Ganguly S., Zernicka-Goetz M. (2013). Asymmetric localization of Cdx2 mRNA during the first cell-fate decision in early mouse development. Cell Rep..

[bib28] Stringer E.J., Duluc I., Saandi T., Davidson I., Bialecka M., Sato T., Barker N., Clevers H., Pritchard C.A., Winton D.J. (2012). Cdx2 determines the fate of postnatal intestinal endoderm. Development.

[bib29] Strumpf D., Mao C.A., Yamanaka Y., Ralston A., Chawengsaksophak K., Beck F., Rossant J. (2005). Cdx2 is required for correct cell fate specification and differentiation of trophectoderm in the mouse blastocyst. Development.

[bib30] Tarkowski A.K., Wroblewska J. (1967). Development of blastomeres of mouse eggs isolated at the 4- and 8-cell stage. J. Embryol. Exp. Morphol..

[bib31] Thomas F.C., Sheth B., Eckert J.J., Bazzoni G., Dejana E., Fleming T.P. (2004). Contribution of JAM-1 to epithelial differentiation and tight-junction biogenesis in the mouse preimplantation embryo. J. Cell Sci..

[bib32] Truett G.E., Heeger P., Mynatt R.L., Truett A.A., Walker J.A., Warman M.L. (2000). Preparation of PCR-quality mouse genomic DNA with hot sodium hydroxide and tris (HotSHOT). Biotechniques.

[bib33] Van Blerkom J. (1981). Structural relationships and posttranslational modification of stage-specific proteins synthesized during preimplantation development of the mouse. Proc. Natl. Acad. Sci. USA.

[bib34] van Rooijen C., Simmini S., Bialecka M., Neijts R., van de Ven C., Beck F., Deschamps J. (2012). Evolutionarily conserved requirement of Cdx for post-occipital tissue emergence. Development.

[bib35] Vinot S., Le T., Ohno S., Pawson T., Maro B., Louvet-Vallee S. (2005). Asymmetric distribution of PAR proteins in the mouse embryo begins at the 8-cell stage during compaction. Dev. Biol..

[bib36] Wu G., Gentile L., Fuchikami T., Sutter J., Psathaki K., Esteves T.C., Araúzo-Bravo M.J., Ortmeier C., Verberk G., Abe K., Schöler H.R. (2010). Initiation of trophectoderm lineage specification in mouse embryos is independent of Cdx2. Development.

[bib37] Zhao T., Gan Q., Stokes A., Lassiter R.N., Wang Y., Chan J., Han J.X., Pleasure D.E., Epstein J.A., Zhou C.J. (2014). β-catenin regulates Pax3 and Cdx2 for caudal neural tube closure and elongation. Development.

[bib38] Zuccotti M., Merico V., Bellone M., Mulas F., Sacchi L., Rebuzzini P., Prigione A., Redi C.A., Bellazzi R., Adjaye J., Garagna S. (2011). Gatekeeper of pluripotency: a common Oct4 transcriptional network operates in mouse eggs and embryonic stem cells. BMC Genomics.

